# Estimating disease burden of a potential A(H7N9) pandemic influenza outbreak in the United States

**DOI:** 10.1186/s12889-017-4884-5

**Published:** 2017-11-25

**Authors:** Walter Silva, Tapas K. Das, Ricardo Izurieta

**Affiliations:** 1 Department of Industrial and Management System Engineering, University of South Florida, Tampa, FL 33620 USA; 2 College of Public Health, University of South Florida, Tampa, FL 33620 USA

**Keywords:** Influenza, Influenza a virus -H7N9 subtype, Agent-based simulation model, Cluster analysis, Sampling studies

## Abstract

**Background:**

Since spring 2013, periodic emergence of avian influenza A(H7N9) virus in China has heightened the concern for a possible pandemic outbreak among humans, though it is believed that the virus is not yet human-to-human transmittable. Till June 2017, A(H7N9) has resulted in 1533 laboratory-confirmed cases of human infections causing 592 deaths. The aim of this paper is to present disease burden estimates (measured by infection attack rates (IAR) and number of deaths) in the event of a possible pandemic outbreak caused by human-to-human transmission capability acquired by A(H7N9) virus. Even though such a pandemic will likely spread worldwide, our focus in this paper is to estimate the impact on the United States alone.

**Method:**

The method first uses a data clustering technique to divide 50 states in the U.S. into a small number of clusters. Thereafter, for a few selected states in each cluster, the method employs an agent-based (AB) model to simulate human A(H7N9) influenza pandemic outbreaks. The model uses demographic and epidemiological data. A few selected non-pharmaceutical intervention (NPI) measures are applied to mitigate the outbreaks. Disease burden for the U.S. is estimated by combining results from the clusters applying a method used in stratified sampling.

**Results:**

Two possible pandemic scenarios with *R*_0_ = 1.5 and 1.8 are examined. Infection attack rates with 95% C.I. (Confidence Interval) for *R*_0_ = 1.5 and 1.8 are estimated to be 18.78% (17.3–20.27) and 25.05% (23.11–26.99), respectively. The corresponding number of deaths (95% C.I.), per 100,000, are 7252.3 (6598.45–7907.33) and 9670.99 (8953.66–10,389.95).

**Conclusions:**

The results reflect a possible worst-case scenario where the outbreak extends over all states of the U.S. and antivirals and vaccines are not administered. Our disease burden estimations are also likely to be somewhat high due to the fact that only dense urban regions covering approximately 3% of the geographic area and 81% of the population are used for simulating sample outbreaks. Outcomes from these simulations are extrapolated over the remaining 19% of the population spread sparsely over 97% of the area. Furthermore, the full extent of possible NPIs, if deployed, could also have lowered the disease burden estimates.

## Background

**Fig. 1:**
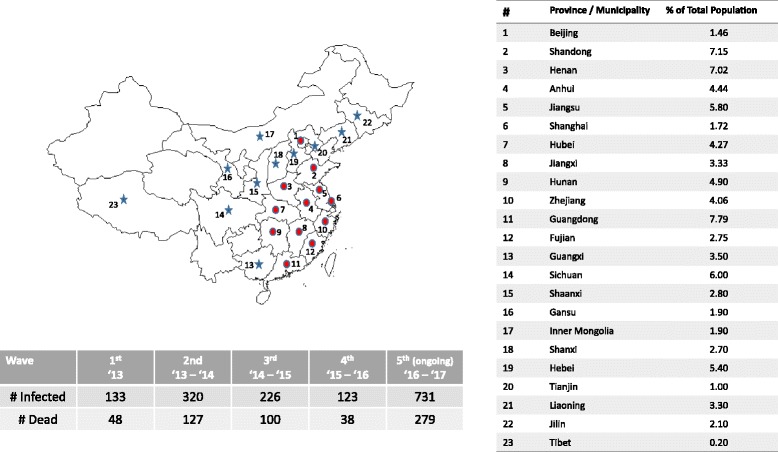
Extent and impact of waves of A(H7N9) outbreaks in China

A(H7N9) has infected humans in China in four waves, spring 2013, winter – spring of 2013–14, 2014–15, and 2015–2016. The fifth wave is currently in progress. As of June 2017, a total of 1533 laboratory-confirmed cases of A(H7N9) infections have been recorded in China causing 592 deaths [1]. The fifth (ongoing) wave has by far been the most widespread covering 23 provinces compared to 12 affected provinces till the fourth wave. It has infected almost the same number of people as the total of all four previous waves combined. Figure 1 depicts the outbreak locations and the numbers of infected and dead, for which information was collected from all applicable WHO reports on A(H7N9). We note that the affected provinces are relatively densely populated regions of China with over 84% of the population. The map in Fig. 1 was generated using the *mapdata* library from *R* software. Though most of the infections are known to be isolated cases, exceptions were noted where human-to-human transmission may have occurred. For example, there were at least 16 clusters of three infected family members and one cluster of two infected family members [2]. However, there is still lack of sustained evidence of human-to-human transmission [1].

A similar situation existed during the years 2003–2009 when the experts believed that a potential pandemic outbreak could be triggered by the H5N1 strain of the influenza virus. As during that period, H5N1 virus infected a total of 468 people with 282 deaths in 15 countries [3]. These numbers were updated in 2015 to 844 infected and 449 deaths [4]. Reports from critical examination of the impact of potential H5N1 outbreaks were presented to the literature in years 2005 [5] and 2006 [6]. Unexpectedly, however, instead of H5N1, the A(H1N1)pdm09 strain caused a worldwide influenza pandemic in 2009. This produced 60.8 million infections and 12,469 deaths in the U.S. alone [7].

Experts fear that A(H7N9) could become human-to-human transmittable and cause a pandemic. Hence, from a public health preparedness standpoint, it is essential to be able to assess the possible impact (disease burden) of a A(H7N9) pandemic. This paper addresses this need by developing a general methodology and applying it on a specific case concerning a pandemic covering all 50 states of the U.S.

Study of the data gathered from A(H7N9) infections reported in [8–14] present some of the characteristic epidemiological parameters for the virus. We have used these parameters in our disease burden estimation model. We note though that since A(H7N9) is not yet human-to-human transmittable, the actual parameters in the event of a pandemic may be different. Consequently, the true outcome of a pandemic may differ from the results presented in our paper. However, our AB simulation model and the estimation methodology are not affected by this limitation. As the new parameters are available for human transmittable scenarios, the model can be rerun and burden estimates can de refined. A comparison of A(H7N9) parameters with those for H5N1, both obtained from animal-to-human transmission cases, is shown in Table 1 [15]. A(H7N9) has been found so far to cause mild to severe disease in humans. In birds, generally, A(H7N9) has been low pathogenic (i.e., it did not cause clinical disease). However, recent observations suggest that the A(H7N9) virus has undergone some changes and it may increase its pathogenicity [16].

**Table 1:** ^b^Comparative parameters for H5N1 and A(H7N9)

Characteristic	H5N1	A(H7N9)
Incubation (days)	3.3 ± 1.5	3.1 ± 1.4
Latent Period (days)	2.15	< 3
Fatality risk	70%(*China*)	32%(*China*)
^a^Admission to death	5.7 *days*	12 *days*
^a^Admission to discharge	18.7 *days*	41. 7 *days*
^a^Median Age	26	62
^a^Poultry exposure	71%	75%

^a^Presented for information only; not used in our model

^b^The numbers are obtained from [15]

In what follows, we explain our methodology for estimating disease burden (measured by IAR and number of deaths) on the U.S. assuming that A(H7N9) becomes human-to-human transmittable and causes a pandemic. Our method is founded on an agent-based simulation model that replicates the dynamics of the social and viral behavior during a pandemic. The simulation model being computing intensive, we apply it selectively on a few sample states in the U.S. for this purpose, all 50 states of the U.S. were first subdivided into smaller sub-groups using a clustering technique. Sample states for simulation are then chosen from each sub-group. A statistical method is used for calculating overall disease burden from the sampled data.

## Methods

Agent-based (AB) simulation is a useful tool to emulate events that might occur in the future and thus support policy makers to prepare measures to address such events. Our AB simulation model incorporates four basic components: demographic information, human behavior, epidemiological characteristics of the virus, and non-pharmaceutical interventions (NPIs) to mitigate pandemic impact.

The demographic information includes the household composition (age, sex, work, and parental status), schools, workplaces, and communities. The human behavior describes the contact process in the mixing groups, compliance to quarantine, and isolation, and travel behavior. The epidemiological characteristics include the disease natural history, parameters affecting the force of infection, basic reproduction number (R_0_), and the fatality rate. Selection of R_0_ values was guided by similar studies (See “Model Validation” section). The NPIs are explained in details in “Non-Pharmaceutical Intervention” section.

Our AB simulation model is quite granular and uses a large computer memory. In its present form, the model is limited to run with up to five million people in an outbreak region. Given this limitation, in order to implement our methodology for a countrywide outbreak in the U.S. with 307 million people, we used a sampling approach. We divided the group of all 50 states of the U.S. into smaller clusters (sub-groups) of states. The clustering technique used to divide the states in smaller sub-groups considered urban population size and density (pop/mile^2^) as attributes of the states. These attributes are highly correlated to the spread of human influenza virus. We applied the simulation model in a few selected states from each sub-group of states. Results from sample states are used to obtain disease burden for the sub-groups, which are then combined to estimate disease burden for the whole country.

### AB simulation model

We used a previous version of our AB simulation model that was presented in [17–21]. We modified the model by incorporating a more detailed method for estimating the probability of infection for a susceptible using the measure of *force of infection* as defined in [5]. The force of infection measures the total daily viral load gathered by a susceptible individual from the infected contacts in the mixing groups. (More details for force of infection are presented in “Infection Model” section).

The model mimics the contact process and tracks each individual in an outbreak region using their scheduled hourly movements within the mixing groups: households, places (schools and workplaces), and community locations.

The model begins by generating the simulated individuals according to the U.S. census and demographic data [22] that gives population attributes including age, gender, and occupational status (school/work). Thereafter, we generate the households based on their composition (characterized by the number of adults and children) in the U.S. (see Tables 2 and 3). We randomly assign each individual to a household while maintaining the average household composition. We then generate the schools, workplaces, and other community locations. We assign each individual a daily (hour by hour) schedule, chosen randomly from a set of alternative schedules based on their attributes. The schedules also vary between weekdays and weekends. Simulation begins on the day when one or more infected people are introduced to the region (referred to as day 1). Simulation model tracks hourly movements of each individual (susceptible and infected) throughout the day, and records for each susceptible the number of contacts with infected at each location. At the end of each day, the model calculates for each susceptible the total amount of viral load ingested (force of infection) from all contacts during that day. The severity of infection of the infected contacts and the place of contact (household, schools, workplaces, and community locations) play critical roles in determining the force of infection. This is used in calculating the probability of infection. The model updates the infection status of all individuals to account for new infections and disease progressions of the already infected ones. The key components of the AB model are described next.

**Table 2:** U.S. Household composition per census 2014

Household Composition		
# adults	# children	Proportion
1	0	28%
1	1	4%
2	0	31%
1	2	4%
2	1	13%
1	3	1%
2	2	13%
1	4	1%
2	3	6%

**Table 3:** U.S. Age distribution per census 2014

Age distribution of household members			
Children		Adults	
Age range	Proportion	Age range	Proportion
[0 − 5]	24%	[23 − 29]	16%
[6 − 9]	23%	[30 − 64]	67%
[10 − 14]	25%	[65+	17%
[15 − 17]	13%		
[18 − 22]	15%		

#### Disease natural history

We adopted a similar disease natural history model was used in previous studies for other influenza A virus strains [5, 19]. Though an accurate disease natural history for human-to-human transmittable A(H7N9) virus is not yet known, we were guided by the disease natural history of other influenza viruses that have already caused pandemic outbreaks.

**Fig. 2:**
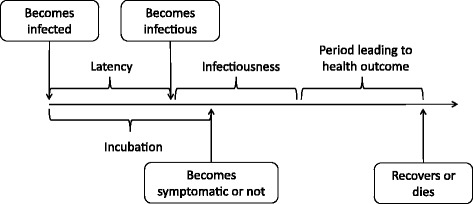
Schematic for the disease natural history

Figure 2 presents a schematic for the disease natural history. An infected individual simultaneously begins a latency and an incubation period (based on parameters given in Table 1). The individual displays symptoms (unless asymptomatic) at the end of the incubation period, and becomes infectious after the latent period is complete. Following the infectiousness period, an infected either recovers or dies. The numerical values of the parameters characterizing the various elements of the disease natural history (e.g., length of incubation period, death rate) are given in Tables 1 and 4. Those who recover are considered to attain sterilizing immunity to further infections [23]. We made this assumption as we are not aware of the immune response to the A(H7N9) virus. Also, as no estimate is available for the proportion of asymptomatic cases for A(H7N9) infections, we assumed it to be 50%. Same proportion of asymptomatic cases was considered in previous studies [5]. The duration of infectiousness for each case is considered to be guided by a lognormal random distribution.

**Table 4:** Parameter values used in the AB simulation model

Parameter	Values
*β*_*h*_	0.47/*day* (for *R*_0_ = 1.8); 0.39/*day* (for *R*_0_ = 1.5)
${\beta}_p^j$	0.94/*day* for schools and 0.47/*day* for workplaces (for *R*_0_ = 1.8); 0.78/*day* for schools and 0.39/*day* for workplaces (for *R*_0_ = 1.5)
*β*_*c*_	0.075/*day* (for *R*_0_ = 1.8); 0.06/*day* (for *R*_0_ = 1.5);
*κ*(*t* − *τ*_*k*_)	lognormal distribution with: *δ* = − 0.72 and *γ* = 1.8
${\psi}_p^j\left(t-{\tau}_k\right)$	0.2 (for schools) and 0.5 (for workplaces) only when the elapsed time since the onset of infection is greater than the latent period 0.25 days; the value of ${\psi}_p^j$ is 0 otherwise
*f*(*d*_*i*_, *k*)	$\frac{1}{{\left[1+\frac{d_i,k}{a}\right]}^b},$Z with *a =* 35 *km* and *b* = 6.5
*ζ*(*a*_*i*_)	*ζ*(*a*_*i*_) = 100% if age ∈[20 − 65], 75% if age ∈[15 − 20] and [65 − 70], 50% if age ∈[10 − 15] and [70 − 75], 25% if age ∈[5 − 10] and [75 − 85], 0% if age ∈[0 − 5]
*ρ*_*k*_	1
*C*_*k*_	1 if individual *k* is a severe infection, 0 otherwise
*ω*	2
${n}_i^{\alpha }$	obtained from the households generated by the model
*α*	0.8
${m}_i^j$	obtained from the population and places generated by the model
death rate	38.61%
symptomatic	50%

#### Infection model

An individual *i* is considered to accumulate force of infection *λ*_*i*_ in his/her home, places, and community locations. It is calculated using the following expression as given in [5].

1 $$ {\displaystyle \begin{array}{c}{\lambda}_i=\sum \limits_{\left.k\right|{h}_k={h}_i}\frac{I_k{\beta}_h\kappa \left(t-{\tau}_k\right){\rho}_k\left[1+{C}_k\left(\omega -1\right)\right]}{n_i^{\alpha }}\\ {}+\sum \limits_{j,\left.k\right|{l}_k^j={l}_i^j}\frac{I_k{\beta}_p^j\kappa \left(t-{\tau}_k\right){\rho}_k\left[1+{C}_k\left({\omega \psi}_p^j\left(t-{\tau}_k\right)-1\right)\right]}{m_i^j}\\ {}+\frac{\sum_k{I}_k\varsigma \left({a}_i\right){\beta}_c\kappa \left(t-{\tau}_k\right){\rho}_kf\left({d}_{i,k}\right)\left[1+{C}_k\left(\omega -1\right)\right]}{\sum_kf\left({d}_{i,k}\right)}.\end{array}} $$

The first component in (1) expresses the force experienced by susceptible individual *i* at home from other infected household members *k*. The second component captures the force experienced at places (schools and workplaces) when a susceptible *i* is in the same place as infected *k*. The third component considers the force of infection gathered from all infected members of the community visited by susceptible *i*. The parameters of (1) are defined in Table 5. *λ*_*i*_ is calculated at the end of each day for all susceptible *i* and the probability of infection is obtained as $1-{\mathit{\exp}}^{-{\lambda}_i}$. It is assumed that if not infected by the end of a day, *λ*_*i*_ is reset to zero. That is, the force of infection is assumed to not accumulate from 1 day to the next.

**Table 5:** Parameters used in calculating the force of infection

Parameter	Description
*I*_*k*_	1 if infected and 0 otherwise
*β*_*h*_	household transmission parameter
${\beta}_p^j$	place transmission parameter
*β*_*c*_	community transmission parameter
*κ*(*t* − *τ*_*k*_)	infectiousness at time (*t* − *τ*_*k*_) since infection
${\psi}_p^j\left(t-{\tau}_k\right)$	factor by which within-place contact rates change for symptomatic severe infection (reflecting sickness-induced absenteeism)
*f*(*d*_*i*_, *k*)	a function of distance *d*_*i*,*k*_ between individuals *i* and *k*
*ζ*(*a*_*i*_)	relative travel-related contact rate of an individual of age *a*_*i*_
*ρ*_*k*_	relative infectiousness of individual *k*
*C*_*k*_	1 if infection is severe, 0 for mild (asymptomatic)
*ω*	2, infectiousness of a severe infection relative to a mild one
${n}_i^{\alpha }$	number of people in the household of individual *i*
*α*	power that determines the scaling of household transmission rates with household size
${m}_i^j$	number of people in the place type *j*

#### Non-pharmaceutical intervention

We considered isolation of symptomatic infected individuals at home for a specific duration with isolation compliance of 53% for adult workers and 57.5% for non-workers [19]. A compliant infected individual is assumed to stay home all day. We consider an isolation threshold of 1 day (that is, on average an individual diagnosed with infection does not begin isolation until the day after) and isolation duration of 7 days.

We also considered household quarantine that restricts the movement of susceptible household members when one or more members are infected. Household quarantine parameters were considered same as for individual isolation. Children were assumed to fully comply with isolation. A partial school closure approach is considered. A classroom is closed when a threshold of newly infected children in the classroom is reached. A threshold for the number of closed classrooms is used to close a school. We used a threshold value of one for both the classroom closure and school closure, and 21 days for the length of school closure. Workplace closure strategy was similar to that of school closure, with each department/group treated like a classroom. The thresholds were: three cases to close a department, 30% of the departments closed to close a workplace, and 7 days for closure duration.

### Clustering technique

**Fig. 3:**
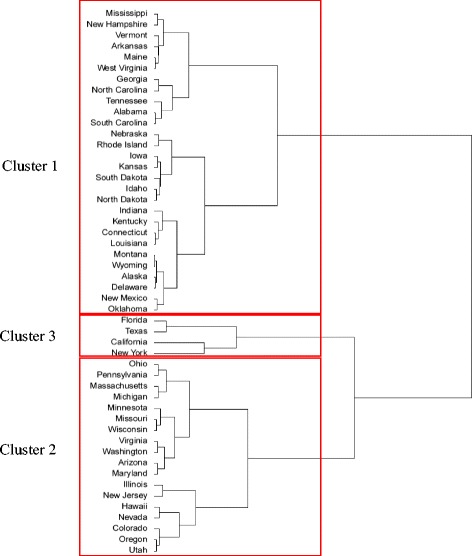
Dendrogram with the list of states contained within three clusters

We adopted a commonly used hierarchical clustering (grouping) technique [24]. The clustering technique forms sub-groups of states such that the chosen attributes of the states within a sub-group are similar to one another and at the same time dissimilar from the attributes in other sub-groups. Higher the level of similarity within a sub-group and dissimilarity between sub-groups, better is the sub-group formation. A clustering method is hierarchical when it creates a set of nested sub-groups that are organized as a tree. At the lowest level of such a tree, each state is separate sub-group, and at the highest level, all states belong to one sub-group (see for example, Fig. 3). The user decides the number of sub-groups (clusters) to consider based on a desired level of similarity form the modeling application.

As stated earlier, we chose urban population size and population density (per square mile) as the two attributes of the states in the U.S. to be used by the clustering technique. We focused on urban population for two reasons: 1) urban areas are more prone to pandemic spread, and 2) states with large population exceeded our simulation model capacity.

The attribute values were first normalized by subtracting from each the corresponding mean and dividing by the corresponding standard deviation. Such a normalization is generally recommended when the numerical values of one or more of the attributes vary significantly within the set. For example, in the U.S., the urban population sizes of the states vary between 1.62 and 36.8 millions, while the urban density ranges between 0.0019 and 0.0043 millions/sq. mile.

The clustering method begins by assigning each state to a separate cluster resulting in 50 clusters at start. Then it calculates the Euclidean distance between the attribute vectors (size, density) of all cluster pairs. (Euclidean distance is the length of the straight line joining two points in the two dimensional space representing the state pair.) The method then identifies the cluster pair with the smallest distance and combines them into one cluster. This reduces the number of original clusters by one, and for the combined cluster, its attribute vector is assigned as the centroid of the attribute vectors of the constituent states. At the next step, the distances between the clusters are updated, and the process repeats until all clusters are combined into one single cluster. A line diagram (called dendrogram; see, for example, Fig. 3) is then used in selecting an acceptable number of clusters considering a desired level of similarity within each cluster. We implemented the clustering method by first using *preprocess* function within the *Caret* package of *R* library to normalize the data. Thereafter, we used the *predict*, *dist*, and *hclust* functions within the *stats* package of *R* library for the remaining steps of the hierarchical clustering method.

### Disease burden calculation from estimates stratified by cluster and age-groups

We first calculated the mean and C.I. of the infection attack rates (IAR), for all three age-groups (indexed by *a*) in all sampled states (indexed by *i*) within each cluster (indexed by *j*), using replicated results of the AB simulation model with different seeds for the random variables. The 100(1 − *α*)% C.I. for IAR was calculated as $$ {\widehat{p}}_{ij}^a\pm {t}_{\raisebox{1ex}{$\alpha $}\!\left/ \!\raisebox{-1ex}{$2$}\right.,n-1}\frac{s}{\sqrt{n}} $$, where $$ {\widehat{p}}_{ij}^a $$ denotes the mean IAR, *n* represents the number of simulation replicates, and *s* represents the standard deviation of the replicated IAR estimates. Mean IAR values for the clusters were obtained by combining the values of $$ {\widehat{p}}_{ij}^a $$into $$ {\widehat{p}}_j^a $$ using the expression below [24].

2 $$ {\widehat{p}}_j^a=\frac{1}{n_j^a}\sum \limits_{i=1}^{S_j}{n}_{ij}^a{\widehat{p}}_{ij}^a, $$

where *S*_*j*_ denotes the total number of selected states that were simulated within cluster *j*, and $$ {n}_{ij}^a $$ represents the size of the urban population in state *i* within cluster *j* for age-group *a*. Note that $$ {n}_j^a={\sum}_{i=1}^{S_j}{n}_{ij}^a $$ is the total urban population for the selected states in cluster *j* for age-group *a*. The 100(1 − *α*)%C.I. on the IAR estimate for each age group within a cluster was obtained as $$ {\widehat{p}}_j^a\pm {t}_{\raisebox{1ex}{$\alpha $}\!\left/ \!\raisebox{-1ex}{$2$}\right.,n-1}\frac{s_j^a}{\sqrt{n}} $$ . The pooled standard deviation $$ {s}_j^a $$ was calculated from the estimates of standard deviation for each selected state within a cluster as the square root of $$ \left[\left(n-1\right){s}_{j1}^{a^2}+\dots +\left(n-1\right){s}_{jk}^{a^2}\right]/\left[k\left(n-1\right)\right] $$, where *k* is the number of selected states in cluster *j*, *n* is the number of simulation replicates, and $$ {s}_{jk}^a $$ is the standard deviation of replicated IAR estimates for age-group *a* in state *k* within cluster *j*. Hereafter, we combined the IAR estimates from all clusters into one value for each age-group using

3 $$ {\widehat{p}}^a=\frac{1}{N^a}\sum \limits_{j=1}^C{N}_j^a{\widehat{p}}_j^a, $$

where *C* denotes the number of clusters, ${N}_j^a$ denotes the total population of all the states in cluster *j* in age-group *a*, and *N* ^*a*^ denotes the total population in the country in age-group *a*. It may be noted that the estimate ${\widehat{p}}^a$ has a variance *V* ^*a*^ from two sources of variability: 1) due to sampling: a sample population from each cluster is used to estimate ${\widehat{p}}_j^a$, which is then assumed to hold good for the whole cluster population, 2) due to simulation based estimation: ${\widehat{p}}_j^a$’s are obtained from ${\widehat{p}}_{ij}^a$, which are estimated from simulation model with inherent variability. It can be argued that these two sources of variability are independent. We obtained the variance due to sampling ${V}_1^a$ as follows [25],

4 $$ {V}_1^a=\frac{1}{{\left({N}^a\right)}^2}\sum \limits_{j=1}^C{\left({N}_j^a\right)}^2\left(\frac{N_j^a-{n}_j^a}{N_j^a}\right)\left(\frac{{\widehat{p}}_j^a\left(1-{\widehat{p}}_j^a\right)}{n_j^a-1}\right). $$

The variance due to simulation $$ {V}_2^a $$ was obtained as the pooled variance from the variance estimates ($$ {s}_j^a $$) of the three clusters as $$ {V}_2^a=\left[\Big(\left(n-1\right){s}_1^{a^2}+\left(n-1\right){s}_2^{a^2}+\left(n-1\right){s}_3^{a^2}\right]/\left[3\left(n-1\right)\right] $$, where *n* is number of simulation replicates per cluster. A 100(1 − *α*)%C.I. was calculated as $$ {\widehat{p}}^a\pm {t}_{\raisebox{1ex}{$\alpha $}\!\left/ \!\raisebox{-1ex}{$2$}\right.,n-1}\sqrt{\raisebox{1ex}{${V}^a$}\!\left/ \!\raisebox{-1ex}{$n$}\right.} $$, where$$ {V}^a={V}_1^a+{V}_2^a $$. Finally, we obtained a single estimate of IAR ($$ \widehat{p} $$) for the whole U.S. across all age-groups *a ϵ*{1, 2, …, *L*} using (3) and substituting in this equation *N*, *N* ^*a*^, and $$ {\widehat{p}}^a $$ for $$ {N}^a,{N}_j^a $$, and $$ {\widehat{p}}_j^a $$, respectively, and summing over *a* = 1 through *L*. The variance *V* on the overall IAR estimate was obtained by pooling variance values *V* ^*a*^ from the three age-groups. A 100(1 − *α*)% C.I. on IAR was calculated as $$ \widehat{p}\pm {t}_{\raisebox{1ex}{$\alpha $}\!\left/ \!\raisebox{-1ex}{$2$}\right.,n-1}\sqrt{\raisebox{1ex}{$V$}\!\left/ \!\raisebox{-1ex}{$n$}\right.} $$ . The number of deaths for each age group and also for the whole U.S. were calculated by applying the death rate on the corresponding numbers for infected persons.

### Model validation

We validated our model by using it to replicate a H5N1 outbreak study for Southeast Asia [5]. Our choice of this validation approach was motivated by similarities between the H5N1 and A(H7N9) virus strains. Both of these strains have still not acquired human-to-human transmission capability, but experts fear that they may mutate to that state. Also our modeling approach has much in common with that used in [5], and hence it provided an appropriate platform for validation. The study considered a population size of 85 million. We considered a subset of the population (5 M) and proportionately adjusted down the number of households, schools, workplaces, and community locations. As in [5], we considered two different cases of *R*_0_ (1.5 and 1.8) values. We used ten replicates for each case, and did not deploy any NPIs since these were not used in [5]. For *R*_0_ = 1.5, the average IAR is 34.58% with a standard deviation of 5.24, and 95% C.I. of [30.83–38.33]. The IAR reported in [5] for *R*_0_ = 1.5 is 33%, which is within our C.I. Our corresponding results for *R*_0_ = 1.8 are 55.7%, 8.16, and [49.86–61.54], respectively. The IAR reported in [5] is 50%. We note that in both cases of *R*_0_ values, results reported in [5] lie in the lower half of our C.I.s.

## Results

**Fig. 4:**
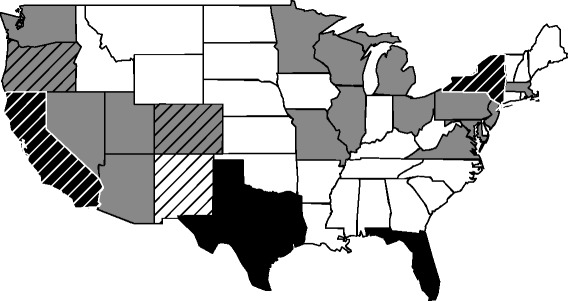
Map of 48 states of U.S. designated to clusters 1(*white*), 2(*gray*), 3(*black*). States marked with lines were selected for outbreak simulation. Not shown in the figure are Alaska (cluster 1) and Hawaii (cluster 2)

The dendrogram in Fig. 3 shows the outcome of clustering of the 50 states of the U.S. into sub-groups using population size and density as the characteristic features (attributes). Based on the selection criteria of the dendrogram, possible choices were either two or three clusters. We chose the three cluster option, which provided better similarity within the clusters; higher similarity was manifested by lower standard deviation of the attribute values within the clusters. We labelled the clusters with numbers 1, 2, and 3, which are composed of states with low, medium, and high values of the attributes, respectively. Figure 4 is a map of U.S. that depicts the cluster designation of all the states. For simulating sample outbreaks, we selected New Mexico from cluster 1, Colorado and Oregon from cluster 2, and California and New York from cluster 3. Selection of these five states was influenced by a recent paper [26] that presented disease burden estimates for seasonal influenza outbreaks in the U.S. However, choice of these particular states from the three clusters does not present a limitation of our study.

For outbreaks in California and New York with urban population sizes greater than five million, we selected a number of contiguous urban counties within each state with a cumulative population of up to five million. While for Colorado, Oregon, and New Mexico we simulated their total urban population, each being less than 5 million. Our focus on urban population was guided by the fact that approximately 81% of the population of the selected states reside in dense urban regions constituting on average 3.4% of the land area (see Table 6). We used latest U.S. census data to extract information on households, workplaces, and schools. We implemented an ad-hoc non-pharmaceutical intervention (NPI) strategy comprising measures like isolation, quarantine, school and workplace closures. Pharmaceutical interventions (vaccines and antivirals) were not considered.

**Table 6:** Urban and Rural population distributions in the States selected for simulation

Region	Total pop (M)	Urban pop(*%*)	Urban pop (M)	Urban area(*%*)	Urban density pop/sq. mile
California	38.80	94.95	36.8	5.28	4304
Colorado	5.36	86.15	4.62	1.47	2836
New Mexico	2.09	77.43	1.62	0.68	1929
New York	19.75	87.87	17.35	8.68	4161
Oregon	3.97	81.03	3.22	1.15	2804

As shown in Table 7, the outbreak in the state of Colorado was simulated using the total urban population of 4.62 M as the sample size comprising 1.19 M for ages ≤ 19, 2.83 M for ages 20–64, and 0.59 M for ages 65 and above. This approach was also used for New Mexico and Oregon. For California and New York, we adopted a proportional sampling approach. For example, California has 9.7 M people for age-group ≤ 19 years and 22.4 M and 4.74 M for age-groups 20–64 years and ≥ 65 years, respectively. The AB model used the family composition features from the U.S. census to randomly generate a total of (9.7/36.8)x5M children, (22.4/36.8)x5M adults up to age 64, and (4.74/36.8)x5M adults 65 and above. Also using census data, our model generated a proportional number of households in a region and then populated each household following the average proportion of children and adults in various age-groups in U.S. families as presented in Tables 2 and 3. Thereafter, the model generated the places (schools and workplaces) using census data in [25] and [27], and randomly assigned each individual to a place based on the age-group. Beyond households and places, the model also considered movements of the individuals in the community within the state for daily errands.

**Table 7:** Simulated population size and infection attack rates (IAR)

	Urban population (M)	Sample size (M)	IAR $\left({\widehat{p}}_{ij}^a\right)$ (*R*_0_ = 1.5)	IAR $\left({\widehat{p}}_{ij}^a\right)$ (*R*_0_ = 1.8)
< = 19 yrs				
California	9.7	1.32	0.3272	0.4197
Colorado	1.19	1.19	0.2782	0.3797
New Mexico	0.43	0.43	0.2461	0.3299
New York	4.18	1.21	0.3172	0.4176
Oregon	0.77	0.77	0.2572	0.3777
20 – 64 yrs				
California	22.4	3.04	0.1612	0.2113
Colorado	2.83	2.83	0.1431	0.1906
New Mexico	0.94	0.94	0.1385	0.1856
New York	10.62	3.06	0.1524	0.2007
Oregon	1.93	1.93	0.1419	0.1866
65 + yrs				
California	4.74	0.64	0.1607	0.2291
Colorado	0.59	0.59	0.1447	0.1926
New Mexico	0.25	0.25	0.1248	0.1615
New York	2.55	0.73	0.1589	0.2144
Oregon	0.51	0.51	0.1386	0.1872

The AB model initiated each outbreak by introducing six infected individuals. The parameter values used to calculate the force of infection (*λ*_*i*_) using (1) are shown in Table 4. To calculate the third component of *λ*_*i*_, we assumed, for simplicity, that each day an individual (susceptible or infected who are not compliant with isolation) travel within or outside of their county of residence for errand or leisure. Travel related parameters used in our study were also used in [28].

**Fig. 5:**
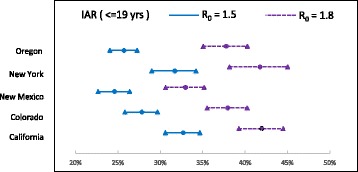
C.I. for infection attack rates for age group ≤ 19 yrs.

**Fig. 6:**
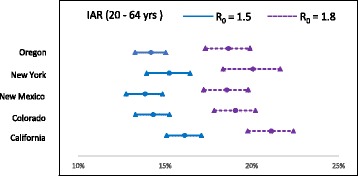
C.I. for infection attack rates for age group 20 - 64 yrs.

**Fig. 7:**
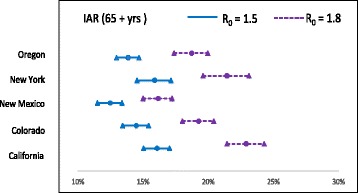
C.I. for infection attack rates for age group 65 + yrs.

The AB simulation model runs (with 10 replicates) in the selected states yielded the mean IAR values (${\widehat{p}}_{ij}^a$) as displayed in Table 7. We used the estimated values of ${\widehat{p}}_{ij}^a$ and ${n}_{ij}^a$ to estimate ${\widehat{p}}_j^a$ using (2), the IARs per age-group within a cluster. These values were then combined to obtain estimate of IAR for each age-group (${\widehat{p}}^a$) in the whole U.S. Finally, IAR values for all age-groups were combined to obtain the overall IAR estimate ($\widehat{p}$). Figures 5, 6 and 7 show the IARs and their C.I.s, which can be seem to be generally higher for states/clusters with higher population density.

Tables 8 and 9 show IARs and number of infected cases per age-group within each cluster. IAR estimates across all clusters within the age-groups (${\widehat{p}}^a$ in (3)) and across all age-groups ($\widehat{p}\Big)$, and the number of deaths are shown in Table 10. It can be observed in Table 10 that IAR for age-group ≤ 19 yrs. is approximately double that of for other two age-groups. Though no age-dependent virus characteristics were considered in the model, the increased IAR among younger population is the result of higher level of social interactions in schools. This can be attributed to the relatively short duration of school closure (21 days) in our NPI strategy.

**Table 8:** Infection attack rates (IAR) per cluster and age-group with 95% C.I

	IAR (in % for R_0_ = 1.5)			IAR (in % for R_0_ = 1.8)		
	Cluster 1	Cluster 2	Cluster 3	Cluster 1	Cluster 2	Cluster 3
< = 19 yrs	24.61	27.00	32.41	32.99	37.89	41.91
	(22.67–26.55)	(25.17–28.83)	(29.98–34.84)	(30.63–35.35)	(35.31–40.47)	(38.74–45.08)
20 – 64 yrs	13.85	14.26	15.84	18.56	18.89	20.79
	(12.76–14.94)	(13.29–15.23)	(14.66–17.02)	(17.23–19.89)	(17.61–20.17)	(19.24–22.34)
65 + yrs	12.48	14.18	16.01	16.15	19.01	22.39
	(11.5–13.46)	(13.21–15.15)	(14.8–17.22)	(14.99–17.31)	(17.72–20.3)	(20.72–24.06)

**Table 9:** Number of infected cases per cluster and age-group with 95% C.I

	Number * (95% CI) R0 = 1.5			Number * (95% CI) R0 = 1.8		
	Cluster 1	Cluster 2	Cluster 3	Cluster 1	Cluster 2	Cluster 3
<=19 yrs	56.49	85.55	132.02	75.73	120.05	170.72
	(52.04–60.95)	(79.75–91.35)	(122.1–141.94)	(70.31–81.15)	(111.89–128.21)	(157.79–183.65)
20–64 yrs	72.22	106.42	79.01	96.77	140.98	103.71
	(66.55–77.88)	(99.18–113.67)	(73.12–84.9)	(89.84–103.71)	(131.41–150.55)	(95.96–111.45)
65+ yrs	16.16	26.15	23.67	20.91	35.06	33.10
	(14.89–17.42)	(24.37–27.93)	(21.88–25.46)	(19.41–22.41)	(32.68–37.44)	(30.63–35.56)

*Numbers in 100,000

**Table 10:** Infection attack rate (IAR), number of infected cases and number of deaths per age-group U.S. with 95% C.I

	R_0_ = 1.5 (95% CI)			R_0_ = 1.8 (95% CI)		
	IAR(%)	# of Infected (million)	Death rate^a^	IAR(%)	# of Infected (million)	Death rate^a^
< = 19 yrs	28.74	27.41	11,094.72	38.43	36.65	14,836.87
	(24.36–33.11)	(23.24–31.58)	(9405.4–12,783.77)	(33.7–43.15)	(32.14–41.16)	(13,011.57–16,660.22)
20 – 64 yrs	14.59	25.77	5631.32	19.33	34.15	7462.97
	(12.05–17.12)	(21.29–30.24)	(4652.51–6610.03)	(16.49–22.17)	(29.13–39.17)	(6366.79–8559.84)
65 + yrs	14.29	6.60	5517.08	19.29	8.91	7447.97
	(9.51–19.06)	(4.39–8.8)	(3671.81–7359.07)	(13.94–24.64)	(6.44–11.38)	(5382.23–9513.5)
Total U.S.	18.78	59.77	7252.30	25.05	79.70	9670.99
	(17.09–20.48)	(54.38–65.16)	(6598.45–7907.33)	(23.19–26.91)	(73.78–85.63)	(8953.66–10,389.95)

^a^Rates per 100,000

It is also observed from Table 10 that the IAR for the two adult age-groups are similar. This is somewhat counterintuitive, as it may be expected that members of age-group 20–64 will have higher IAR resulting from higher work related social interactions. We believe that older people (65 +) are accumulating viral load (force of infection) at a higher rate from age-group 20–64 by being at home with other infected family members.

The IAR estimates were compared with IAR estimates for other viruses as shown in Table 11. Simulation-based estimates of IAR for both H5N1 and A(H7N9) were found to be much lower than the field estimate for H1N1/2009. We conjecture that the lower IAR estimates are due to lower estimates of the force of infection, for which the parameters were estimated from only animal-to-human transmittable cases of outbreaks.

**Table 11:** Comparison of infection attack rates among different influenza viruses

Description	H1N1 2009 (See Ref [7])	H5N1 (See Ref [6])	Seasonal Influenza (See Ref [33, 34])	A(H7N9) (Current Paper)
Data used	surveillance data from U.S. outbreak	simulated outbreak in U.S. and England	Surveillance data from U.S.	Simulated outbreak in U.S.
Method used	Extrapolation with Correction factors	AB simulation model	Proposed by CDC	AB simulation model and stratification
NPIs (school & workplace closure, case isolation)	yes (with antiviral)	yes (with vaccine and antivirals)	no	yes
Age-groups analysis	yes	yes	yes	yes
Estimated IAR	50%	28% for *R*_0_ = 1.7	5% - 10% in adults	18.78% for *R*_0_ = 1.5
		34% for *R*_0_ = 2.0	20% - 30% in children	25.05% for *R*_0_ = 1.8

## Discussion

Our paper is the first to estimate disease burden from A(H7N9) pandemic outbreak, hence we could not directly compare our results with other studies on A(H7N9). The disease parameter estimates used in our model were adopted from the recent reports on epidemiological studies of A(H7N9) [8, 15, 29, 30]. Other models (e.g., using differential equations) have been used to analyze A(H7N9) [30–32]. These models do not have the level of granularity offered by agent-based simulation models. However, such granularity comes with the cost of computation and memory usage, which resulted in our model capacity being limited to 5 million people per simulation run. We note that this limit can be increased with better computing hardware and more efficient usage of memory. Among the five states that were selected for simulation, the size limit only applied to California and New York with urban population sizes much larger than 5 million. We used an age-based proportional sampling approach to select up to 5 million individuals from the urban areas.

A paper published in June 2016 [29] presented a comprehensive analysis of the laboratory-confirmed cases of A(H7N9) infection in mainland China. It offered renewed estimates for incubation period, fatality risk, hospital admission to death/discharge, median age, and poultry exposure. We note, however, that the parameter estimates that we used from an earlier study [15] do not differ significantly from those presented in [29]. Though it appears from the published data that A(H7N9) affects more people of higher age group, it is likely a function of the very high level of poultry exposure (≥ 74% [15]) for the older age group. Our AB model does not incorporate any age-dependent factor for calculating probability of infection. However, our model does consider age-based contact process, which in turn affects the infection probability.

We simulated outbreaks only in urban areas and extrapolated the results to the population in the remaining (rural) areas. Urban areas constitute on average 3.4% of the geographic area and approximately 81% of the population [22]. Hence, the disease burden estimates, for each state, cluster, and age-group, as presented here, are likely to be upper bounds, since the rural areas are likely to yield less number of infections with less contacts and higher distances between individuals. Also, applications of vaccines and antivirals were not considered in our AB model, which increased the number of susceptible and the intensity of infection, respectively. Furthermore, the disease burden estimates could have been lowered by application of the full extent of NPIs.

For the NPI strategy implemented in our simulation model, we chose its parameters (e.g., length of school closure) somewhat arbitrarily. We note that these parameters could be optimized based on the virus and societal characteristics. In another study focused to assess in community resilience for influenza pandemic outbreaks, we tested NPIs with two different sets of parameters for a small A(H7N9) outbreak in a region with 1.1 million people in the state of Florida in U.S. [33]. Table 12 shows the parameters of these NPI strategies and the corresponding IARs. Note that the strategy marked as NPI(1) is same as the strategy used in the study presented in this manuscript, and NPI(2) is the strategy that was recommended in [19].

**Table 12:** NPI parameters

#	Measure	NPI (1)	NPI (2)
1	Global Threshold	10	10
2	Deployment delay	3 days	7 days
3	Case isolation threshold	1 day	1 day
4	Case isolation duration	7 days	10 days
5	Case isolation compliance for workers	75%	75%
6	Case isolation compliance for non-workers	84%	57%
7	Household quarantine threshold	1 day	1 day
8	Household quarantine duration	7 days	7 days
9	Household quarantine compliance workers	75%	53%
10	Household quarantine compliance non-workers	84%	84%
11	Cases to close a class in a school	4	1
12	Classes to close a school	6	3
13	School closure duration	10 days	21 days
14	# cases to close a department in a workplace	6	3
15	% of departments to close a workplace	60%	30%
16	Workplace closure duration	10 days	7 days

## Conclusions

Our AB simulation model has the following notable limitations. It does not assign specific geographic locations for the households, places, and community locations. As a result, we had to use estimated values for the distance between susceptible and infected (*f*(*d*_*i*, *k*_) in Eq. (1)) in calculating the force of infection. Use of antivirals could have reduced the profile of infectiousness with a lower peak and shorter duration, in turn reducing the virus spread and the corresponding IAR. Also, we did not consider pre-existing immunity for any age-group in the population.

We reiterate the fact that A(H7N9) has not yet been found to be human-to-human transmittable. This paper considers the hypothetical scenario of the virus mutating to a state capable of causing a worldwide human pandemic. Also, the disease natural history and the parameters used to calculate force of infection are extrapolated from the data gathered from recent cases of animal-to-human transmissions of A(H7N9) as well as data from previous pandemics caused by related viruses. Hence, the numbers presented in this paper are estimates at best.

## Abbreviations

AB: Agent-based
IAR: Infection attack rate
NPI: Non-pharmaceutical intervention
